# Patterns of Welfare Attitudes in the Australian Population

**DOI:** 10.1371/journal.pone.0142792

**Published:** 2015-11-10

**Authors:** Timothy P. Schofield, Peter Butterworth

**Affiliations:** Centre for Research on Ageing, Health & Wellbeing, Research School of Population Health, The Australian National University, Canberra, Australian Capital Territory, Australia; University of New South Wales, AUSTRALIA

## Abstract

The study of community attitudes toward welfare and welfare recipients is an area of increasing interest. This is not only because negative attitudes can lead to stigmatization and discrimination, but because of the relevance of social attitudes to policy decisions. We quantify the attitudes toward welfare in the Australian population using attitude data from a nationally representative survey (*N* = 3243). Although there was broad support for the social welfare system, negative attitudes are held toward those who receive welfare benefits. Using canonical correlation analysis we identify multivariate associations between welfare attitudes and respondent demographic characteristics. A primary attitudinal dimension of welfare positivity was found amongst those with higher levels of education, life instability, and personal exposure to the welfare system. Other patterns of negative welfare attitudes appeared to be motivated by beliefs that the respondent’s personal circumstances indicate their deservingness. Moreover, a previously unidentified and unconsidered subset of respondents was identified. This group had positive attitudes toward receiving government benefits despite having no recent experience of welfare. They did, however, possess many of the characteristics that frequently lead to welfare receipt. These results provide insights into not only how attitudinal patterns segment across the population, but are of relevance to policy makers considering how to align welfare reform with community attitudes.

## Introduction

The income support (or welfare) system is a key element of Australia’s social safety net, providing financial assistance to the most vulnerable and disadvantaged members of the community [1,2]. Although Australian welfare payments are means tested, a relatively large portion (around a quarter, 27%) of the population are receiving income support payments at any point in time [3]. Moreover, this is true of 17% of the working age population [4]. In addition to the goal of providing support for a basic standard of living for all, Australian welfare policy is increasingly used to encourage self-sufficiency, promote social and economic participation, and enhance personal and family wellbeing [1]. These positive policy goals may be thwarted if the Australian community views the welfare system and those receiving benefits with suspicion and contempt. Thus, we contend there is a need to investigate and consider the patterns and consequences of such community attitudes within the Australian context. The present work focuses on the multivariate clustering of attitudes across the community.

A process of welfare reform has been underway in Australia since the 1990s [5]. Recent changes aimed at reducing welfare dependence and increasing workforce participation have included extending waiting times for some unemployment claimants and introducing work requirements for recipients of disability payments [6]. A further tranche of reform options were outlined in the Interim Report of the Reference Group for Welfare Reform released in 2014. These policy directions were said to respond to concerns the Australian welfare system “does not reflect the values, expectations and day-to-day experiences of the Australian community” (pg. 27). This statement implies a necessarily strong and direct relationship between public attitudes and welfare policy, but there is scant information about the attitudes of Australians towards the welfare system and its beneficiaries. There is, therefore, a need to systematically investigate Australian community attitudes to welfare.

Investigating attitudes towards welfare is also an important first step in understanding the causes and consequences of welfare stigma. The presence of negative attitudes is one of the four pre-requisite conditions for stigma identified by Link and Phelan [7] in their comprehensive theoretical framework of stigma (i.e., the discrediting of someone or some group on the basis of labelling an undesirable characteristic [8,9]). According to Link and Phelan [7] stigmatization occurs when the differentiating characteristic is labelled; the characteristic has negative associations; the characteristic leads to separation from society; and those possessing the characteristic are discriminated against. Welfare stigma is important to understand as it may be used as a policy level to deter inappropriate take-up of welfare payments [10] and minimize individuals’ exposure to moral hazards such as dependency and fraud [11]. However, the existing research evidence suggests it may be the most disadvantaged who are most likely to be deterred if welfare receipt is stigmatized [12]. Further, although direct evidence of stigma in the domain of welfare is lacking, the potential for stigma to generate adverse health and psychological outcomes [7,13] requires further consideration. If stigma is present it may be a contributing factor to the poorer physical and mental health of welfare recipients [14,15]. Thus, the current study of community attitudes to welfare and recipients also represents the starting point for research into the broader manifestation of welfare stigma (and its consequences) in Australia.

### Attitudes to welfare

Individuals can have disparate attitudes toward the welfare system and welfare recipients. For instance, individuals may be supportive of the scope of government welfare policy but be unhappy with its efficiency [16]. There is considerable cross-national variation in both attitudes toward welfare systems [16] and welfare recipients [17]. While there is considerable cultural variability, there also seems to be some consistency in that a deservingness heuristic drives attitudes: the perceptions of who is deserving of support may differ from country to country [18], but the underlying impact of perceived deservingness is consistent [19]. As perceptions of who is deserving may change over time, data on changes in attitudes to welfare form an important part of the empirical backdrop. In Britain, where public attitudes to welfare have been monitored over decades, data show: a substantial decline in support for increased spending on welfare benefits for the poor; that an increasing majority consider benefits for the unemployed are too generous; and that there are growing levels of distrust and concern about the integrity of the welfare system [17,20,21]. Moreover, recent European data shows Britain to have the most negative attitudes toward welfare recipients, as evident from strong endorsement of the view that they are “lazy and dependent” [17].

Much less is known about the attitudes of Australians to welfare. The little work that has taken place has largely focussed on the structure of the welfare system, demonstrating broad support for its mutual obligation (reciprocity) framework [22] (e.g., active engagement in seeking work, training or community activity in exchange for welfare benefits). While there is little direct quantification of Australian’s attitudes toward welfare recipients, we anticipate strongly negative attitudes given analysis of the language of welfare recipients as “dole bludgers” [23], and widespread negative media coverage exemplified by headlines such as “stop the bludgers” [24].

While the first objective of this study was to quantify Australian attitudes towards welfare recipients, the second objective was to investigate the diversity of welfare attitudes across different subpopulations. International research suggests those with lower levels of educational attainment and in poorer socio-economic circumstances may hold more negative attitudes, while the personal experience of welfare receipt may ameliorate these attitudes [11]. We adopted a data-driven multivariate approach to understand how welfare attitudes are segmented across the Australian population. Multivariate approaches have been used previously, but have been focussed on the internal structures of attitudes, rather than their structure across different segments of the population [16]. Moreover, the focus of such analysis has been largely on attitudes toward the welfare system, rather than welfare recipients.

The objective of the current study, is to investigate the prevalence, patterns and profiles of welfare attitudes in Australia. The project examines negative attitudes to create an empirical backdrop that may help in discussions of attitude-driven welfare reform in Australia, but the results have relevance to other countries undergoing welfare system reform. The study reports analysis of nationally representative survey data which measures welfare attitudes via respondent endorsement of a series of statements describing attitudes and beliefs about welfare benefits, welfare payments and welfare recipients. In this way, the measures capture attitudes toward both welfare recipients and the welfare system. These items had been drawn from the pool of items used in the European Social Survey (2008) and the New Zealand Election Study (2005 and 2008), which will help to facilitate international comparison. The first aim of the current study is to document the extent to which the Australian population hold negative welfare attitudes. Given the considerable cross-national variation in welfare attitudes [17] it is important to add this information to the broader literature. The second aim is more unique and involves using the technique of canonical correlation analysis (CCA) to identify and describe the characteristics of (statistically salient) groups in the population who hold distinct views of social benefits and welfare recipients. CCA is a multivariate clustering technique that uses information from within the outcome items (like, principal components/factor analysis), but also draws in information external to the outcome (like, predictors in a multiple regression). This approach represents a new direction for the field and one we contend facilitates deeper insight into the potential causes and patterns of welfare attitudes, and may produce an important new evidence base for policy makers.

## Methods

### Data

This study reports analysis of data from a cross-sectional, nationally representative survey of the Australian population; the 2009 iteration of the Australian Survey of Social Attitudes (AuSSA) [25]. This is third party data and was not collected by the authors. AuSSA 2009 received ethics approval from the Australian National University. The AuSSA has been conducted since 2003 and is a biennial mail survey of Australians randomly selected from the Electoral Roll. With a few minor restrictions, enrolment on the Electoral Roll is compulsory for adult Australians, and individuals can enrol from age 17. Individuals enrolled with no fixed address or with their address redacted due to safety concerns would not be contactable. Moreover, some individuals with dementia or that have been deemed incapable of understanding the electoral process may be removed from the roll.

Two versions of AuSSA 2009 were distributed at random, Version A had a response rate of 37% (*n* = 1718) and Version B a rate of 33% (*n* = 1525); both versions included the measures of interest (total *N* = 3243). The publically available 2009 dataset was selected because it was the most recent iteration of AuSSA which included questions assessing attitudes toward welfare receipt and recipients. Valid data on each of the nine key attitudinal items were available for between 89.4% and 95.1% of respondents, with only 89 respondents (2.7%) not responding to any of the welfare attitude items. To correct for the potential of sampling and respondent bias in the sample, weights included with the data set were applied. The use of these weights ensure that population estimates derived from statistical models more closely reflect the Australian population. The authors accessed the AuSSA data via the Australian Data Archive (https://www.ada.edu.au/). The data is open access on registration with further information regarding measures and sampling available.

### Measures/items

Nine items assessing welfare attitudes were present in AuSSA 2009, these items were rated by respondents on a 5 point scale from strongly agree to strongly disagree. These responses were recoded for analysis such that higher scores indicate more negative attitudes toward welfare and lower scores less negative attitudes (see Table 1). While some of these statements are more about the welfare system, e.g., “cutting welfare benefits would damage too many people’s lives”, and its scope, e.g., “All families deserve payments from the government to help with the costs of raising children”, “single parents deserve government payments so they can be home to raise their children”, the remainder are focused upon the individuals within the system.

**Table 1:** Attitudinal items collected in AuSSA 2009.

Original Statement	Descriptive Label
Cutting welfare benefits would damage too many people’s lives.	*Disagree* cuts damage lives
People who receive welfare benefits should be under more obligation to find work.	More obligation to work
It is too hard to qualify for welfare benefits in Australia today	*Disagree* too hard to qualify
All families deserve payments from the government to help with the costs of raising children.	*Disagree* families deserve
Around here most unemployed people could find a job if they really wanted to.	Easy to find a job
Welfare benefits make people lazy and dependent	Lazy and dependent
Most people getting welfare benefits are trying to find a job.	*Disagree* most trying to find job
The government should limit the length of time that people can get welfare benefits even if they end up without an income.	Should limit time on welfare
Single parents deserve government payments so they can be home to raise their children.	*Disagree* single parents deserve

Welfare items were rated from 1 (strongly agree) to 5 (strongly disagree), and subsequently recoded for analyses such that higher scores indicate more negative attitudes toward welfare, and lower scores indicate less negative attitudes.

A range of demographic measures were used to investigate the segmentation of attitudes across the population. These demographics were sex, age, educational attainment, housing stability, household structure, current employment status (unemployment), and prior welfare exposure (Table 2). Prior welfare exposure is defined as either the respondent or their partner receiving some form of government benefit in the last 5 years, the levels of other variables are noted in Table 2. A direct measure of household income was not used in these analyses due to high rates of missingness, however, recent prior exposure to welfare payments serves as a suitable marker of low household income [26]. The heavy targeting and tight income and assets tests for welfare benefits in Australia makes welfare receipt a strong correlate of low household income.

**Table 2:** Demographic characteristics of the samples.

	Full Sample	Full Sample	CCA Sample
	Weighted	Unweighted	Unweighted
**Education**			
Bachelor or above *	18.63%	28.42%	27.00%
Less than year 12 *	33.19%	19.80%	18.74%
**Renting** *	34.10%	23.91%	24.20%
**Partnered** *	62.10%	68.49%	69.46%
**Dependent children** *			
0	72.43%	67.38%	66.63%
1	10.34%	11.72%	12.58%
2	10.88%	13.14%	13.97%
3+	5.02%	6.09%	6.82%
**Age** *			
17–34	35.54%	17.74%	18.91%
35–49	14.81%	23.49%	24.58%
50–64	25.26%	33.86%	35.47%
65+	23.38%	23.11%	21.04%
**Male** *	48.41%	44.65%	45.91%
**Unemployed** *	2.44%	1.75%	1.80%
**Exposure to benefits** *	52.67%	51.79%	51.66%

The characteristics used in each analysis are presented as percent non-missing data.

Note

* indicates that the variable was included in the CCA model

### Statistical approach/Analysis strategy

After presentation of key demographic characteristics of the sample, two sets of analyses are reported. The first analysis addresses Aim 1 and presents the weighted prevalence of negative welfare attitudes in Australia. In addition, effect sizes and one-sample *t*-tests quantify the strength of negative attitudes relative to neutral attitudes.

The second set of analyses use canonical correlation analysis (CCA) to address Aim 2, examining how welfare attitudes vary across demographic segments of the population. CCA differs from other clustering techniques used in psychology (e.g., factor analysis) in that it draws in information from both the outcome variable set and the predictor set, simultaneously, to determine the clusters. A simpler approach would have been be to conduct separate regression analyses for each attitudinal item separately. However, this approach would inflate type 1 error rates. Moreover, it would not capture covariation among attitudinal items. Alternatively, if the attitudinal items are simply summed, or even analysed via principal components analysis, and the covariates regressed on the overall welfare attitude measure(s) then type 1 error is controlled, but potentially important multi-dimensional patterning within the dependent attitude measures would be missed. Therefore, a canonical correlation approach was used to capture the multi-dimensional nature of these attitudinal variables in relation to demographic characteristics.

In brief, CCA takes a set of independent variables (X_1_…X_i_) and dependent variables (Y_1_…Y_i_) and weights the variables within each set to create two maximally correlated variates (for a guide to CCA see [27]). The correlation between these two variates is the canonical correlation (R_c_), and is interpreted analogously to Pearson R. This process is repeated within the unexplained variance to create a set of orthogonal canonical correlations. The maximum number of canonical correlations in this set is equal to the smaller number of X or Y variables. Each canonical correlation explains a unique subset of variance between the X and Y variates as they are orthogonal to each other canonical correlation in the set. A restricted subset of canonical correlations which represented statistically significant (*p* < .05) and meaningful (R_c_ > 0.1) correlations were selected for interpretation. Five canonical functions met this criteria. To investigate the utility of this approach, the canonical functions were compared to the components identified from an initial principal components analysis.

The standardized canonical function coefficients (coef.; similar to standardized beta coefficients in regression), structure coefficients (r_s_; similar to factor loadings in principle components analysis) and canonical cross-loadings (cros.; also called redundancy coefficients; the relationship between a variable in one set and the other variate) were examined to aid interpretation of the 5 canonical correlation functions. Positive standardized canonical function coefficients and structure coefficients on the attitude measures indicate more negative welfare attitudes, whereas for the demographic features they indicate more of the attribute (either a yes response for binary variables or more of the characteristic for ordinal variables). When the attitude variate was associated with prior receipt of welfare benefits, follow-up correlation analyses were conducted between participant’s scores on the computed attitude variate and the raw welfare receipt items. These follow-up analyses investigate which form of welfare experience is likely associated with the welfare attitude profile. As neither STATA nor SPSS supports the use of sample weights in CCA, by necessity it was conducted, on an unweighted dataset, with cases with missing data omitted. Preliminary descriptive analyses are presented to confirm the similarity of the sample used in CCA analysis to the broader sample and population (i.e., weighted sample).

## Results

### Preliminary

Characteristics of the full 3241 respondents (1447 females) are presented in Table 2, along with the sample characteristics after weighting. Approximately 50% of the sample had either received or had their partner receive some form of welfare benefit in the preceding 5 years.

### Quantification of welfare attitudes

**Fig 1:**
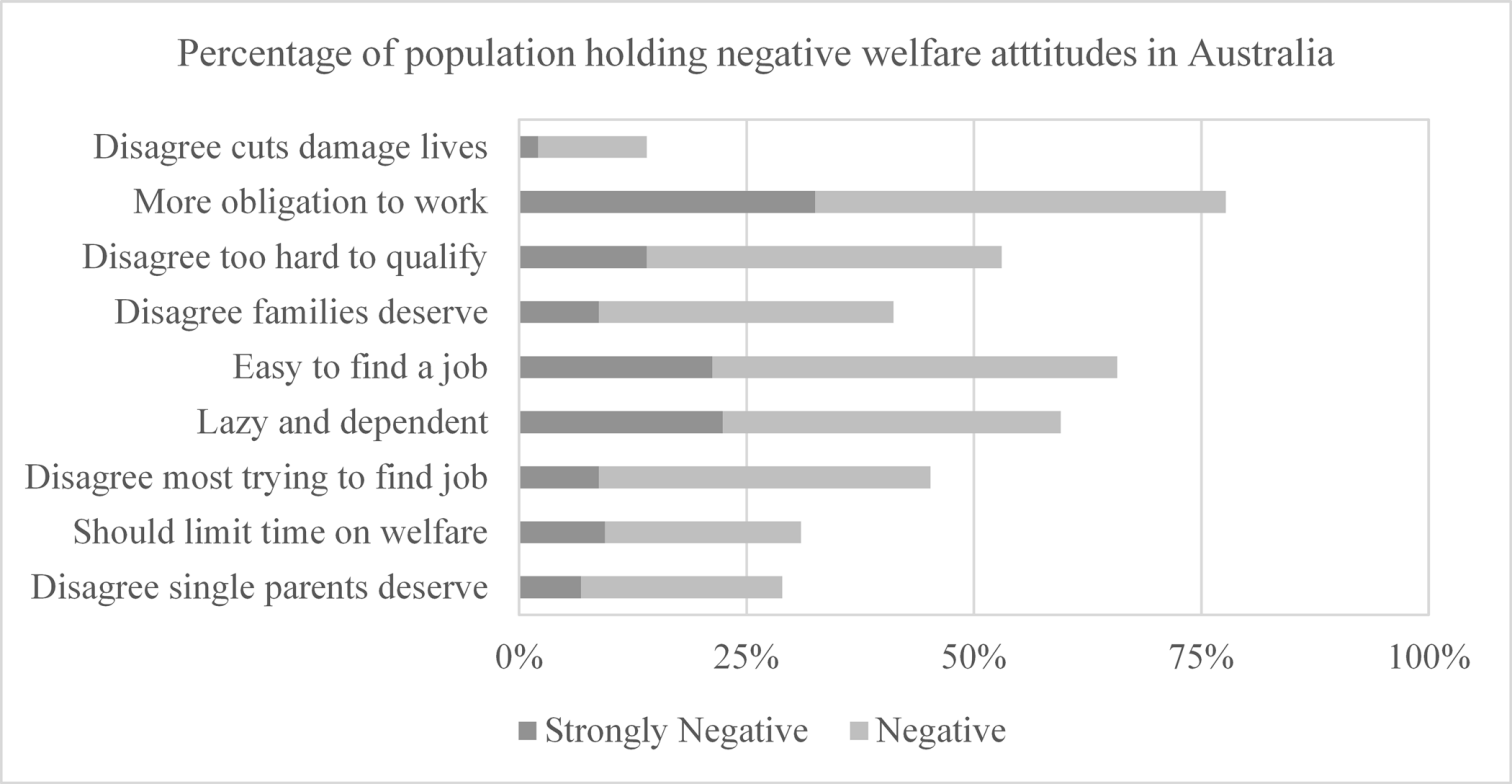
Prevalence of negative welfare attitude responses. Five levels of response to each statement were given to respondents for each statement; strongly agree, agree, neither agree nor disagree, disagree, strongly disagree. Darker bars indicate the percentage of strongly negative responses, light bars indicate the percentage of negative responses.

The negativity of attitudes toward welfare and welfare recipients are presented in Fig 1. This figure, and the statistical results evident in Table 3, show that only a minority of respondents held negative views of the welfare system as indicated by few individuals disagreeing with the beliefs that cuts damage lives and that welfare should be time limited. Attitudes towards the availability of welfare payments for families, including single parents, were positive but weak. In contrast, the population held much stronger negative attitudes towards welfare recipients themselves. The majority of respondents held the view that there is need for recipients to have greater obligations to look for work, that recipients could find jobs if they really wanted to, that welfare benefits make people lazy and dependent, that it is too easy to qualify for welfare benefits, and that most people on welfare benefits are not trying to find work.

**Table 3:** Tests of attitude valence.

	Weighted one-sample t-test							Sensitivity test		
Attitude	df	t		d	95% CI			Weighted MI		
Disagree cuts damage lives	3051.19	46.38	***	0.84	0.82	to	0.89	0.81	to	0.89
More obligation to work	3082.00	-58.42	***	1.05	-1.03	to	-0.97	-1.03	to	-0.96
Disagree too hard to qualify	2898.37	-22.66	***	0.42	-0.48	to	-0.40	-0.47	to	-0.40
Disagree families deserve	3075.76	3.24	**	0.06	0.03	to	0.11	0.03	to	0.12
Easy to find a job	3071.28	-33.76	***	0.61	-0.69	to	-0.61	-0.69	to	-0.61
Lazy and dependent	3070.90	-30.94	***	0.56	-0.65	to	-0.57	-0.64	to	-0.57
Disagree most trying to find job	2935.39	-14.54	***	0.27	-0.30	to	-0.23	-0.29	to	-0.21
Should limit time on welfare	3025.17	9.52	***	0.17	0.16	to	0.25	0.16	to	0.24
Disagree single parents deserve	3037.98	9.32	***	0.17	0.15	to	0.23	0.15	to	0.23

Outcomes of sample-weighted one sample t-tests against a value of 3 (indicating a neutral response). Cohen’s d is reported as a measure of effect size. Comparison confidence intervals are presented for an imputed data set (assumption of missing at random) using sample weighted multiple imputation. Imputation was performed in SPSS v22 using MCMC with seed set to 3319607 and a maximum of 10 iterations and 5 imputed datasets.

*** denotes that the effect is significant at p < .001

** denotes that the effect is significant at p < .01.

### Segmentation of attitudes

We first performed a principal components analysis of the welfare attitude items. This is a multivariate analysis of attitude patterns not taking into account the multivariate patterning of the individuals providing these attitudes, as CCA would. Two components had eigenvalues over 1, accounting for 53.09% of the variance in the attitudinal items. The first component was broadly one of negative attitudes toward welfare recipients, while the second component was broadly one of negative attitudes toward parenting payments (Table 4). The correlations of these principal components with the *orthogonal* canonical attitude variates are discussed later to highlight the explanatory utility of the CCA approach.

**Table 4:** Results of Principal components analysis.

	Component 1	Component 2
	Negativity to the welfare system and welfare recipients	Negativity toward parenting payments and eligibility
*Disagree* cuts damage lives	0.333	0.161
More obligation to work	0.374	-0.157
*Disagree* too hard to qualify	0.255	0.386
*Disagree* families deserve	0.099	0.652
Easy to find a job	0.372	-0.308
Lazy and dependent	0.436	-0.215
*Disagree* most trying to find work	0.366	0.035
Limit time on welfare	0.384	-0.195
*Disagree* single parents deserve	0.254	0.442

Unrotated component loadings from principal component analysis of attitudes.

*Note*: Loadings < |.2| are not reported.

The demographic characteristics of the 2433 respondents with no missing data w included in the CCA analyses are also presented alongside those for the full sample in Table 2. It is evident that the sample used in the CCA analyses does not differ appreciably from the full sample, suggesting that there is unlikely to be systematic bias.

In the current CCA analysis, the full canonical model was significant, λ(81, 15616) = 8.257, *p* < .001. The first five canonical correlations represent statistically significant small to medium correlations, and were interpreted. The next 3 canonical functions were very small (R_c_ < .1) and so, although statistically significant, are not interpreted. The composition of these canonical correlations is presented in Table 5. To prevent redundancy, in the table R_c_^2^ is reported in percentage points (i.e., from 0 to 100), while in text *R*_*c*_ is reported and presented as a value from 0 to 1. The caption of Table 5 contains further information about standardized canonical function coefficients and canonical cross-loadings.

**Table 5:** Canonical solution for demographics predicting attitudes toward welfare for canonical functions 1–5.

	*Function 1*			*Function 2*			*Function 3*			*Function 4*			*Function 5*			
*Variable*	*Coef*	*Cros*.	*r*_*s*_	*Coef*	*Cros*.	*r*_*s*_	*Coef*	*Cros*.	*r*_*s*_	*Coef*	*Cros*.	*r*_*s*_	*Coef*	*Cros*.	*r*_*s*_	*h* ^*2*^ *(%)*
*Disagree* cuts damage lives	-0.384	-0.220	**-0.700**	-0.896	-0.156	-0.598	-0.529	-0.048	-0.212	-0.058	-0.003	-0.022	0.108	-0.010	-0.102	90.34
More obligation to work	-0.325	-0.224	**-0.710**	0.077	0.063	0.243	0.002	0.011	0.050	0.344	0.033	0.216	0.065	-0.008	-0.081	61.89
*Disagree* too hard to qualify	0.180	-0.065	-0.206	0.005	-0.029	-0.113	0.321	0.087	0.385	0.575	0.060	0.394	-0.736	-0.073	-0.711	86.33
*Disagree* families deserve	0.009	-0.031	-0.099	-0.157	-0.079	-0.302	0.443	0.140	0.623	-0.851	-0.093	-0.606	-0.203	-0.026	-0.250	91.87
Easy to find a job	-0.239	-0.207	-0.658	0.319	0.119	0.457	-0.295	-0.037	-0.165	-0.081	-0.009	-0.059	-0.124	-0.016	-0.151	69.56
Lazy and dependent	-0.076	-0.224	-0.711	0.432	0.104	0.400	0.250	0.015	0.068	-0.342	-0.021	-0.136	0.401	0.000	-0.004	68.80
*Disagree* most trying to find work	-0.155	-0.187	-0.594	-0.048	0.012	0.047	-0.206	-0.005	-0.024	-0.085	-0.013	-0.083	-0.464	-0.045	-0.440	55.59
Limit time on welfare	-0.137	-0.203	-0.646	0.243	0.066	0.251	0.086	-0.003	-0.014	-0.192	-0.022	-0.145	-0.273	-0.026	-0.255	56.67
*Disagree* single parents deserve	-0.272	-0.170	-0.540	-0.108	-0.051	-0.196	0.633	0.149	0.661	0.430	0.034	0.225	0.554	0.028	0.274	89.28
R^2^_c_ (%)			9.90			6.80			5.05			2.34			1.05	
Bachelor degree plus	0.566	0.136	0.433	-0.642	-0.212	-0.811	0.146	-0.001	-0.004	-0.123	-0.017	-0.111	0.117	-0.001	-0.008	85.70
Did not finish school	-0.017	-0.017	-0.055	0.410	0.164	0.630	0.010	0.045	0.199	-0.185	-0.007	-0.048	0.368	0.032	0.315	54.07
Renting	0.282	0.120	0.381	0.065	0.048	0.183	-0.162	-0.093	-0.414	-0.033	-0.059	-0.384	0.713	0.047	0.455	70.50
Live with partner	-0.270	-0.136	-0.432	-0.107	-0.065	-0.250	0.018	0.008	0.035	0.226	0.075	0.487	-0.130	-0.018	-0.175	51.81
Dependent child	-0.138	-0.053	-0.169	-0.140	-0.045	-0.172	-0.373	-0.120	-0.535	0.715	0.111	0.724	0.591	0.015	0.142	88.83
Age	0.256	0.023	0.072	-0.082	0.046	0.175	0.767	0.201	0.897	0.552	0.056	0.364	0.529	0.015	0.142	99.27
Male	-0.173	-0.061	-0.193	-0.013	-0.004	-0.017	0.041	0.032	0.143	-0.188	-0.017	-0.113	0.237	0.024	0.231	12.42
Unemployed	0.278	0.113	0.359	-0.034	0.001	0.005	-0.107	-0.043	-0.190	0.192	0.018	0.120	0.327	0.030	0.288	26.20
Exposure to benefits	0.608	0.184	0.585	0.379	0.119	0.457	-0.111	-0.035	-0.155	0.220	0.064	0.420	-0.630	-0.037	-0.365	88.43
*Correlation with principal components*																
Component 1			-0.930			0.130			0.125			-0.003			-0.286	
Component 2			0.074			-0.630			0.723			-0.081			-0.204	

The attitude variate and demographic variate of each function are “latent variables”. The canonical correlation R_c_ is the correlation between the two variates, and the percentage of variance is presented as R^2^_c_ in the middle row of the table. The structure coefficients (r_s_, 3rd column within each function) represent the Pearson correlation between an item and it associated canonical variate as determined by factorization of the correlation matrix (e.g., the correlation of *Disagree* cuts damage lives with the attitudinal variate in the first function is -0.700). Structure coefficients greater than |.35| are underlined, and those greater than |.70| are double underlined. Each individual’s variate scores are calculated by summing the product of the structure coefficient and observed value for each item in the variate (i.e., for attitudes, the items from “*Disagree* cuts damage lives” to “*Disagree* single parents deserve”; for demographics, the items from “Bachelor degree plus” to “exposure to benefits”). The correlation of each item with the alternative variate (e.g., “*Disagree* cuts damage lives” with the demographic variate) is represented in the column of canonical cross loadings (cros., 2^nd^ column within each function). Also presented are the standardized canonical function coefficients (coef., 1^st^ column within each function). These are the standardized β coefficients from simultaneously regressing each item in the variate on the variate itself, and as such can be thought of as adjusted structure coefficients. This adjustment process accounts for their typically smaller size relative to the structure coefficients and their sometimes divergent directions. As the reader moves through the table, the function changes. Each function has its own attitude and demographic variate, and across functions the variates are orthogonal. It is thus useful to know how much of the original items variance is represented by the reported canonical functions, that is, each variables communality (*h* ^*2*^ reported as a %, final column of table). The communality is calculated by taking the sum of squared structure coefficients (r_s_) across the 5 reported functions. Communality coefficients greater than |35.00| are underlined, and those greater than |70.00| are double underlined. The bottom section of the table presents the correlation of the two extracted attitude components from the principle component analysis with the demographic variate of the canonical function.

The first canonical correlation, *R*_*c1*_ = .315 (i.e., *R*_*c*_^*2*^ = 9.90%), *p* < .001, can be best described as an attitude of ‘sympathy to the welfare system and welfare recipients’. This canonical attitude variate was strongly correlated with the first principal component of *‘*negativity to the welfare system and welfare recipients’, *r* = -.930. The attitude variate represents broadly positive views of the welfare system and welfare recipients, paired with ambivalence about whether it is too easy to obtain welfare payments and the deservingness of payments to families. Elevated welfare sympathy was associated with higher education, recent exposure to the welfare system, and general life instability (unpartnered, renting, current unemployment). Follow up analysis suggested that it was primarily exposure to unemployment payments, *r*(2431) = .123, *p* < .001, or disability payments, *r*(2431) = .150, *p* < .001, that were associated with the sympathetic attitude function.

The second canonical correlation, *R*_*c2*_ = .261, p < .001, seems reflective of the view that ‘welfare is important but the people on it are lazy and dependent’. The attitude variate represents a positive view of the welfare system, but a negative view of those in receipt of welfare benefits. This function was predicted by low levels of educational attainment, and recent exposure to the welfare system. Although it may seem counter-intuitive that those with experience of welfare payments hold negative views of welfare recipients, the follow-up analysis suggested that it was primarily exposure to the aged pension, *r*(2431) = .165, *p* < .001, and disability payments, *r*(2431) = .103, *p* < .001, that was responsible for the association with the canonical function. The individuals receiving these payments are largely reliant on the welfare system, but there is little expectation of workforce participation or that they will ever leave their payments. This demographic profile may explain the apparent contradiction in holding views of the importance of the welfare while holding negative views of those benefiting from it.

The third canonical correlation, *R*_*c3*_ = .225, *p* < .001, can be described as a belief that it is ‘too easy to qualify for welfare benefits’. This description was reflected by direct endorsement of this statement and accompanied by negative attitudes toward the deservingness of people in receipt of parenting payments. This attitude was most prevalent in older individuals, those with no dependent children, and those who had more stable housing. The described demographic profile appears to be consistent with a demographic of older working adults and retirees. Consistent with this speculation, exploratory analysis suggested that although these individuals had an experience of welfare generally, they were more likely to have had recent exposure to the aged pension, *r*(2431) = .120, *p* < .001.

Despite the attitude variates of canonical correlations two and three being orthogonal by definition, both were strongly correlated (though in opposite directions) with the second principal component of ‘negativity toward parenting payments and eligibility’, *r*s ≥ |.630|. The fact that this single principal component draws so strongly on two orthogonal population segments indicates the added utility of the CCA approach.

The fourth canonical correlation, *R*_*c4*_ = .153, *p* < .001, is a qualified version of the third function; it is ‘too easy to qualify for welfare benefits, but families do deserve support’. This attitude was most prevalent in older individuals, those who had more stable housing, those who were living with a partner, and those with dependent children. This attitude cluster is also associated with prior exposure to welfare payments, though follow-up analyses suggested this association was confined to receipt of payments for families, *r*(2431) = .069, *p* < .001. A highly targeted and self-serving attitude is suggested by this demographic profile of families with dependent children under some degree of financial strain. The population identified as holding this attitudinal patterning is unrelated to the two attitude principal components extracted. This demonstrates that the subpopulation holding this view of welfare may have been overlooked without the CCA approach adopted here.

The fifth and final interpreted canonical correlation, *R*_*c5*_ = .103, *p* < .001, represents a segment of the population that appears to want a more open and accessible welfare system. The attitude cluster was characterized by a belief that it is too hard to qualify for welfare but that those on welfare payments are trying to find work. The individuals in this cluster were characterized by less stable housing and no prior receipt of welfare benefits themselves.

## Discussion

With welfare reform in Australia described as being driven by the disconnect between public attitudes and the nature of the welfare system, there is a need for research to quantify and profile welfare attitudes across different segments of the population. The present study aims to provide this evidence base. Our analysis suggests that the Australian population holds largely negative views of those receiving welfare benefits. Specifically, we found that welfare recipients were broadly characterized as lazy and not doing enough to find work. These findings in the Australian context are consistent with findings in other English speaking countries [17,28]. While over 60% of those in the UK view welfare as making people lazy and dependent [17], a similar proportion of the present sample endorsed such a negative attitude. This negative view of welfare recipients was accompanied by the belief that recipients should be under more obligation to find work and were not genuinely trying to find a job. Broadly speaking, there is also a perception that it’s too easy to qualify for welfare payments in Australia. The most positive welfare attitudes in Australia were not concerned with the characteristics of the recipients per se, but rather the structure of the welfare system. This same pattern of support for the system but not recipients is also evident in the UK [29]. These negative attitudes toward welfare recipients provide a basis for the stigmatization of welfare recipients according to the Link and Phelan model of stigma [7].

It is important to note that although negative welfare attitudes were present in the community, our innovative application of CCA shows that these attitude clusters are heterogeneous and differ across segments of the population. The CCA analyses broadly suggest that the valence of attitudes toward the welfare system and recipients is linked to levels of education, receipt of stigmatized welfare payments, and life instability. In fact, there was a more general pattern present for education: negative attitudes towards welfare recipients were present among those who were less educated. For the most part, the other profiles of welfare attitudes can be seen as self-serving; those with life stability were not favourably disposed to welfare, and those with children more likely to be positive about payments for families.

Even the unexpected final cluster of individuals that appear to want a more open and accessible welfare system may have been looking out for themselves. This speculation is consistent with their housing vulnerability and the fact that they had not previously benefited from welfare, suggesting their ineligibility. This group may represent the Australian “middle class battler” phenomenon [30], where individuals with sufficient absolute income are left with little disposable income due to excessive spending. Alternatively, it may represent the pressures of living in a country with relatively low levels of housing affordability [31]. Regardless of which of these motivations underpins these views, these individuals may be attracted to welfare in the short term, but–given their belief that welfare recipients are trying to find work–are perhaps only looking to use welfare for a small time period. Short term use of welfare could conceivably help individuals to buffer against peaks in their bills and expenses or rental and mortgage pressures. However, this notion needs direct testing, as no measure of desire to take up welfare benefits was available in the present data. An alternate explanation may be that due to their personal circumstances these individuals recognise the need for a more accessible welfare system but would not move on to it themselves. We speculate that if the individuals in this segment do move on to welfare that they will be less impacted by self-stigma processes [32] as their behaviour is not necessarily incongruent with their own beliefs. However, they may still experience negative outcomes because they anticipate negative treatment due to their awareness of broader societal attitudes [12].

In light of this population segmentation, it becomes pertinent to ask questions about the source and consequences of community-level welfare attitudes. This can build upon the emerging research into structural stigma [33] to investigate the mechanisms through which negative community-level welfare attitudes evolve and their consequences. This can include the impact of welfare receipt on mental health [14,15,34–38].

The present research was restricted to the analysis of items available in the AuSSA dataset, and so could not cover the full breadth of relevant attitudes, or fit neatly within theoretical models [16]. This limitation is offset by the fact that rather than fit the limited items into a broader model, the items were used a data-driven approach. Data-driven handling is not uncommon in this area, as many studies have relied on the same limited arrays of survey items in large nationally representative samples [39]. What distinguishes the present empirical approach is its novel multivariate analytic method. A limitation of the method used to segment attitudes in the population is that CCA did not make use of sample weights. Although not ideal, the inability to use sample weights is not particularly problematic given that CCA examines relationships within the data, and is not used to make population estimates. Despite this limitation, CCA has demonstrated considerable utility over more conventional principal components analysis. Specifically, using this technique we were able to differentiate two distinct population segments that loaded on a single attitudinal principal component. Another limitation of this research is the relatively low response rate to the distributed survey. However, concerns about bias in respondent characteristics are attenuated in two ways. First, the analysis of welfare attitudes make use of sample weights to make demographically unbiased estimates. Second, as the items assessing welfare attitudes are nested within a broader survey of social attitudes, the low response rate is unlikely due to an unwillingness to express their attitudes toward welfare specifically. The limitations are balanced by key strengths of the survey; a relatively large and nationally representative sample.

As in other English speaking countries [17,28], the current study found strong evidence of negative attitudes towards welfare recipients in the Australian population. The negative views of welfare recipients are striking when compared to the relatively strong support for the existence of a welfare system as a social safety net. Moreover, the current study found these views of welfare are not held equally across all segments of the population, nor for each potential recipient group in the population. Future research may find that this is explained as a self-serving belief that one belongs to a group deserving of welfare. The present study provides a concrete evidence base against which policy makers can critically examine whether proposed welfare reform directions indeed reflect community attitudes as has been suggested [1,2], and, perhaps more importantly, identify the segments of society who hold alternate views. Finally, it is important to consider that the patterning of attitudes indicates the plausibility of negative attitudes toward welfare recipients being translated into prejudicial and discriminatory actions. This possibility warrants further direct testing.
